# ¹H NMR-based metabolic profiling of human rectal cancer tissue

**DOI:** 10.1186/1476-4598-12-121

**Published:** 2013-10-18

**Authors:** Huijuan Wang, Liang Wang, Hailong Zhang, Pengchi Deng, Jie Chen, Bin Zhou, Jing Hu, Jun Zou, Wenjie Lu, Pu Xiang, Tianming Wu, Xiaoni Shao, Yuan Li, Zongguang Zhou, Ying-Lan Zhao

**Affiliations:** 1 State Key Laboratory of Biotherapy and Cancer Center, West China Hospital, West China Medical School, Sichuan University, 17#, 3rd Section, Ren min South Road, Chengdu 610041, China; 2 Department of Gastrointestinal surgery, West China Hospital, West China Medical School, Sichuan University, Chengdu 610041, China; 3 Analytical & Testing Center, Sichuan University, Chengdu 610041, China; 4 Department of Pediatric Surgery, West China Hospital, West China Medical School, Sichuan University, Chengdu 610041, China

## Abstract

**Background:**

Rectal cancer is one of the most prevalent tumor types. Understanding the metabolic profile of rectal cancer is important for developing therapeutic approaches and molecular diagnosis.

**Methods:**

Here, we report a metabonomics profiling of tissue samples on a large cohort of human rectal cancer subjects (n = 127) and normal controls (n = 43) using ^1^H nuclear magnetic resonance (^1^H NMR) based metabonomics assay, which is a highly sensitive and non-destructive method for the biomarker identification in biological systems. Principal component analysis (PCA), partial least squares discriminant analysis (PLS-DA) and orthogonal projection to latent structure with discriminant analysis (OPLS-DA) were applied to analyze the ^1^H-NMR profiling data to identify the distinguishing metabolites of rectal cancer.

**Results:**

Excellent separation was obtained and distinguishing metabolites were observed among the different stages of rectal cancer tissues (stage I = 35; stage II = 37; stage III = 37 and stage IV = 18) and normal controls. A total of 38 differential metabolites were identified, 16 of which were closely correlated with the stage of rectal cancer. The up-regulation of 10 metabolites, including lactate, threonine, acetate, glutathione, uracil, succinate, serine, formate, lysine and tyrosine, were detected in the cancer tissues. On the other hand, 6 metabolites, including myo-inositol, taurine, phosphocreatine, creatine, betaine and dimethylglycine were decreased in cancer tissues. These modified metabolites revealed disturbance of energy, amino acids, ketone body and choline metabolism, which may be correlated with the progression of human rectal cancer.

**Conclusion:**

Our findings firstly identify the distinguishing metabolites in different stages of rectal cancer tissues, indicating possibility of the attribution of metabolites disturbance to the progression of rectal cancer. The altered metabolites may be as potential biomarkers, which would provide a promising molecular diagnostic approach for clinical diagnosis of human rectal cancer. The role and underlying mechanism of metabolites in rectal cancer progression are worth being further investigated.

## Introduction

Colorectal cancer (CRC) is the third most frequent malignancy and the fourth most common cause of cancer mortality worldwide [1]. Among CRC, 65% of CRC are rectal cancer, which is located in the lower end of the colon. Although advanced methods of diagnosis such as computed tomography (CT), ultrasonography (US), magnetic resonance imaging (MRI), and treatments such as surgery, neoadjuvant chemotherapy and radiation therapy, have been employed over the last few decades, the overall survival rate of patients with rectal cancer has not improved markedly. Tumor stage has a great influence on survival and is defined by UICC TNM (International Union against Cancer, Tumor Node Metastases) classification. Five-year survival rate of rectal cancer patients is 93.5% for stage I, 87.4% for stage II, 58.2% for stage III, and 8.1% for stage IV [2]. The reasons that result in late diagnosis and therapy as well as disappointingly low survival rate include ineffective screening tools and guidelines, cancer detection at an advanced stage, limited survival achieved with palliative chemotherapy alone for patients with metastatic or unresectable disease. Therefore, early and accurate diagnosis of rectal cancer is critical for patients’ survival and improving therapeutic options for different stages of rectal cancer.

Metabolomics is an emerging field of research downstream of transcriptomics, genomics, and proteomics, which mainly involves the multicomponent analysis of biological fluids, tissues and cell extracts. It is currently used as a model of research in many disciplines of medicine, including disease diagnosis [3,4], biomarker screening [5,6], nutritional intervention [7] and safety assessment of chemical [8,9]. Three powerful analytical techniques are commonly applied to assay and quantify metabolites, including liquid chromatography (LC) coupled with mass spectrometry (MS), gas chromatography MS (GC/MS) and nuclear magnetic resonance (NMR) [10]. NMR has been used extensively since 1970s. It has some advantages over MS in metabolic application, including non-destructive analysis, the relative ease of sample preparation, the potential to identify a broad range of compounds and the capacity for the supply of structural information for unknown compounds [11,12]. Until now, only several NMR-based studies using patient colorectal cancer tissues have been reported [1,13]. However, the number of patient tissues in these studies was limited, which cannot provide accurate and comprehensive information of CRC metabolites. Moreover, discriminating metabolites involved in the different pathological stages of rectal cancer have not been investigated. Therefore, it will be valuable to perform metabolic profiling of human rectal cancer tissues in aiding molecular diagnosis and providing novel insights into rectal cancer.

In the present study, we applied ^1^H-NMR to study metabolic profiling of human rectal cancer tissues and found the metabolic alterations between rectal cancer tissues and normal controls. We identified a total of 38 differential metabolites, 16 of which were closely correlated with the stages of rectal cancer. These modified metabolites potentially revealed disturbance of energy, amino acids, ketone body and choline metabolism in human rectal cancer. Our findings indicate the metabolites disturbance may be associated with the progression of rectal cancer. The altered metabolites may be as potential biomarkers, which would provide a promising molecular diagnostic approach for clinical diagnosis of human rectal cancer. The role and underlying mechanism of metabolites in rectal cancer progression are worth being further investigated.

## Results

### Metabolic profiling of samples

**Figure 1:**
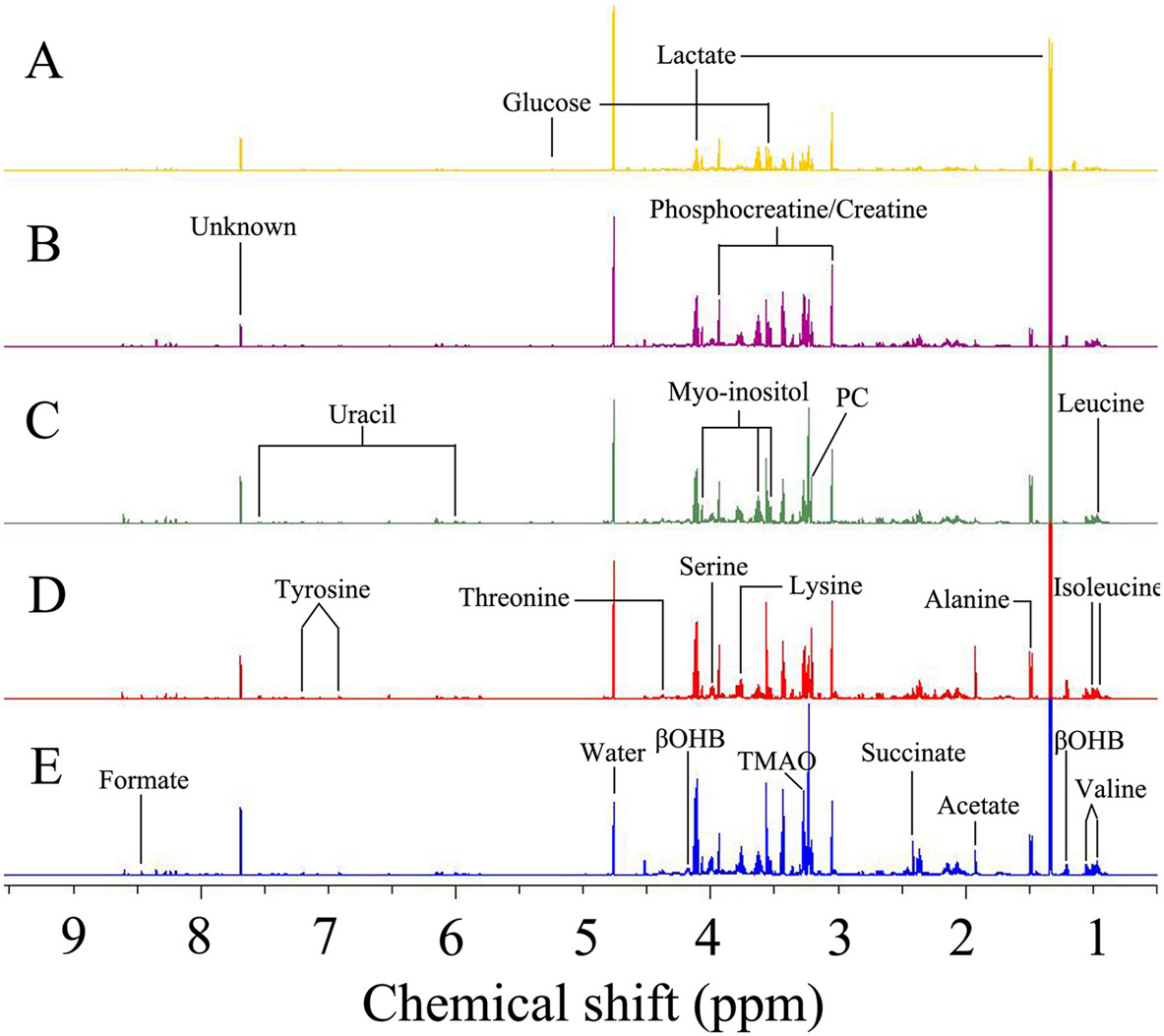
**600 MHz representative **^**1**^**H NMR spectra (δ9.5–δ0.5) of tissue samples. A** normal control, **B** stage I of rectal cancer, **C** stage II of rectal cancer, **D** stage III of rectal cancer, **E** stage IV of rectal cancer.

Typical ^1^H NMR spectra of aqueous phase extracts of rectal cancer tissues of different stages and normal mucosae were shown in Figure 1. The standard one-dimension spectrum gave an overview of all metabolites. The major metabolites in the spectra were identified according to literature data and the Human Metabolome Database (http://www.hmdb.ca/). As a result, a series of changes in endogenous metabolite levels were observed in rectal cancer when compared with the normal mucosa. These metabolites included lactate, threonine, acetate, glutathione, uracil, succinate, serine, formate, lysine, tyrosine, myo-inositol, taurine, phosphocreatine, creatine, betaine, dimethylglycine, which are known to be involved in multiple metabolic processes, especially in energy and amino acid metabolism [14,15].

### PR analysis of normal mucosae and rectal cancer tissues

**Figure 2:**
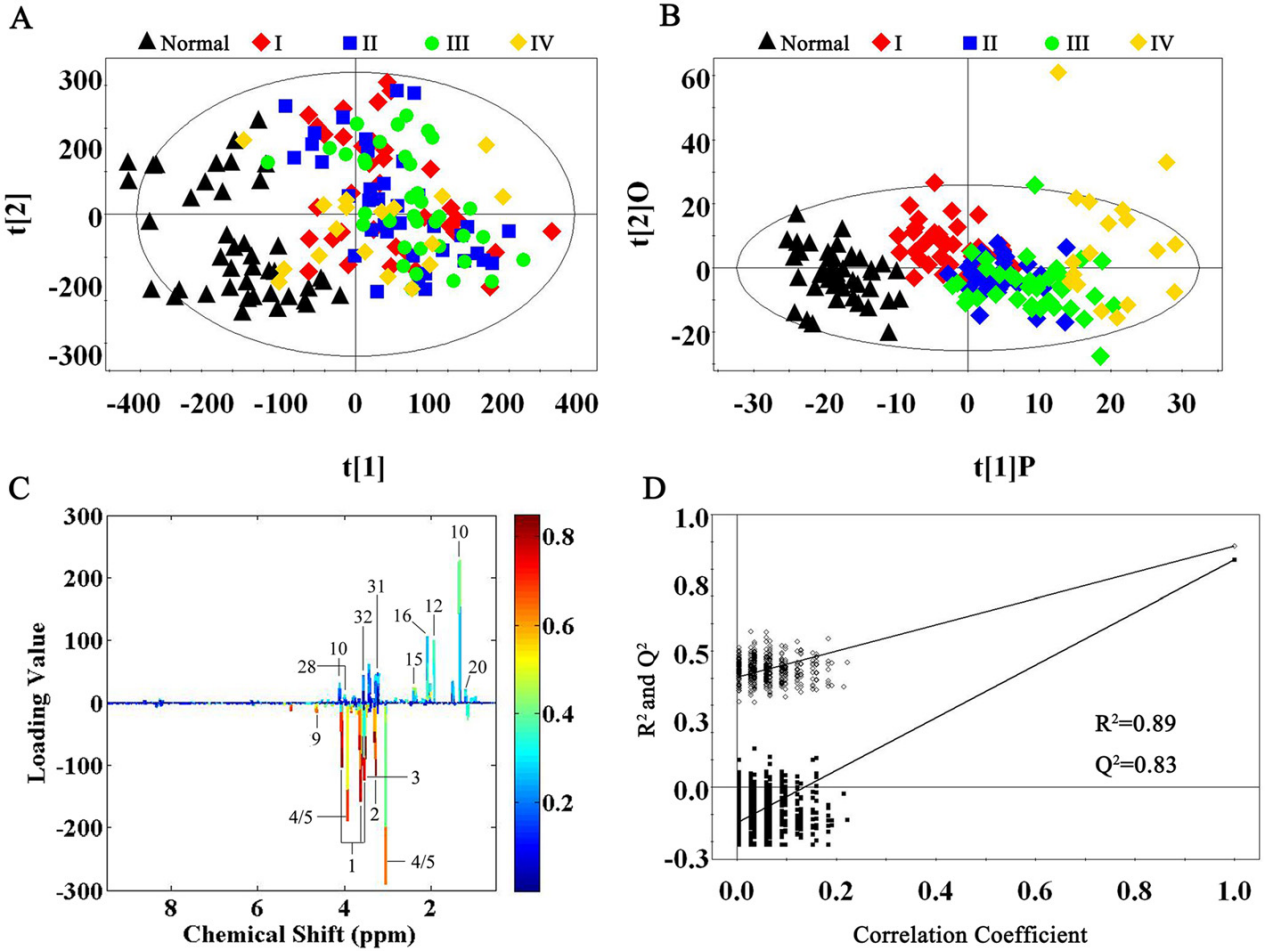
**Metabolite profiles between rectal cancer tissues and normal controls. A** PCA scores plot discriminates metabolites from the rectal cancer tissues and normal controls using ^1^H NMR. **B** OPLS-DA scores plot based on same samples. **C** The color map shows the significance of metabolite variations between the two classes. Peaks in the positive direction indicated the increased metabolites in rectal cancer tissues in comparison to normal control. Deceased metabolites in rectal cancer tissues were presented as peaks in the negative direction. **D** Statistical validation of the corresponding PLS-DA model using permutation analysis (200 times). R^2^ is the explained variance, and Q^2^ is the predictive ability of the model.

To determine the differences between the normal controls and rectal cancer tissues, we initially utilized the PCA to analyze ^1^H NMR data after data normalization. The results showed an apparent separation between rectal cancer tissues and normal controls on the scores plot of first two principal components (PC) (Figure 2A). The majority of samples were located in 95% confidence interval. Therefore, all of samples were used in the following analysis to ensure the maximum information.

To optimize the separation between the rectal cancer tissues and normal controls, of the two groups, we then utilized the OPLS-DA to visualize the metabolic difference. As shown in Figure 2B, good separation in the scores plot of PC1 and PC2 of OPLS-DA analysis was obtained between rectal cancer tissues and normal controls. Moreover, model parameters in the permutation test for the explained variation (R^2^ = 0.89) and the predictive capability (Q^2^ = 0.83) were significantly high, indicating a satisfactory predictive ability (Figure 2D). To identify the main metabolites responsible for the separation between cancer tissues and normal controls, their scores and loadings plots with correlation coefficients were obtained from OPLS-DA analysis based on the NMR data of tissue samples (Figure 2C). The loadings were colored according to the UV model variable weights and showed the significant class-discriminating metabolites responsible for the clustering patterns. The positive signals indicated the up-regulated metabolites in the cancer tissues in comparison with normal controls, including lactate, threonine, acetate, glutathione, uracil, succinate, serine, formate, lysine and tyrosine. On the other hand, the signals in the negative direction indicated the down-regulated metabolites in rectal cancer tissues, including myo-inositol, taurine, phosphocreatine, creatine, betaine and dimethylglycine. The significantly distinguishing metabolites were summarized according to VIP > 1 and *p* < 0.05 (Table 1). According to metabolic pathway on the Kyoto Encyclopedia of Genes and Genomes (KEGG) database (http://www.genome.jp/kegg/), we outlined the main metabolic pathways, which are closely related to rectal cancer morbidity. These metabolic pathways consisted of glycolysis, serine synthesis pathway, TCA cycle, amino acid metabolism, pyrimidine metabolism and gut flora metabolism.

**Table 1:** **Differential Metabolites derived from OPLS-DA model of **^**1**^**H NMR analysis between rectal cancer patients and normal controls**

**Metabolites**	**chemical shift**	**Mutiplicity**^**a**^	**Rectal cancer vs. normal control**		
	**(ppm)**		**VIP**^**b**^	***P*-value**^**c**^	**FC**^**d**^
1 Myo-inositol	4.06	t	4.32	<0.001	-2.00
	3.55	dd	4.21	<0.001	-2.05
	3.63	t	3.91	<0.001	-2.16
2 Taurine	3.27	t	4.25	<0.001	-2.10
3 α-Glucose	3.55	dd	4.21	<0.001	-2.05
	5.23	dd	3.42	<0.001	-4.94
4 Phosphocreatine	3.93	s	3.56	<0.001	-2.13
	3.04	s	3.30	<0.001	-2.11
5 Creatine	3.94	s	3.56	<0.001	-2.13
	3.04	s	3.30	<0.001	-2.11
6 Betaine	3.89	s	2.70	<0.001	-1.89
7 Dimethylglycine	3.71	s	2.64	<0.001	-2.38
8 Glyceryl	4.3	m	2.51	<0.001	-1.45
9 Lactate	1.33	d	2.34	<0.001	1.24
	4.11	q	1.55	<0.001	1.80
10 Threonine	1.33	d	2.34	<0.001	1.24
	4.24	m	0.77	0.296	1.04
11 Acetate	1.93	s	2.27	0.004	2.97
12 Glutathione	2.56	m	2.19	<0.001	1.60
	2.96	m	1.79	0.012	1.23
13 Uracil	7.54	d	2.16	<0.001	3.12
	5.8	d	2.09	<0.001	3.27
14 Succinate	2.41	s	2.14	<0.001	1.84
15 O-acetyl glycoprotein	2.07	s	1.96	<0.001	1.77
16 Dimethylamine	2.73	s	1.94	0.029	1.28
17 Leucine	0.96	t	1.73	<0.001	1.17
18 Valine	1.05	d	1.72	0.166	1.48
	0.99	d	1.66	0.016	1.36
19 β-hydroxybutyrate	1.2	d	1.69	<0.001	2.05
	4.16	m	0.57	0.565	1.04
20 Formate	8.45	s	1.67	0.001	1.43
21 Glutamine	2.14	m	1.63	0.001	1.45
	3.77	m	1.09	<0.001	1.35
22 Acetoacetate	2.28	s	1.55	0.008	1.21
23 Sarcosine	2.75	s	1.48	<0.001	1.02
24 Tyrosine	7.2	d	1.42	<0.001	-1.42
	6.9	d	1.32	0.029	-1.30
25 Alanine	1.48	d	1.38	0.054	1.70
26 Acetoacetic acid	2.31	s	1.36	0.051	1.19
27 Serine	3.98	m	1.29	<0.001	1.45
28 Isoleucine	1.01	d	1.22	0.301	1.13
	0.95	t	1.20	0.002	1.16
29 Methylamine	2.59	s	1.19	0.001	1.30
30 Trimethylamine-N-oxide	3.27	s	1.11	<0.001	2.96
31 Lysine	3.77	m	1.09	<0.001	1.35
32 Acetone	2.23	s	1.06	0.445	1.08
33 PC(phosphochline)	3.21	s	1.05	<0.001	1.22

^a^Multiplicity: *s* singlet, *d* doublet, *t* triplet, *q* quartet, *dd* doublet of doublets, *m* multiplet.

^b^Variable importance in the projection was obtained from OPLS-DA model with a threshold of 1.0.

^c^*p*-value obtained from Student’s t-test.

^d^Fold change(FC) between rectal cancers and normal controls. Fold change with a positive value indicates a relatively higher concentration present in rectal cancer patients while a negative value means a relatively lower concentration as compared to the normal controls.

### PR analysis of between normal mucosae and stage-related rectal cancer tissues

**Figure 3:**
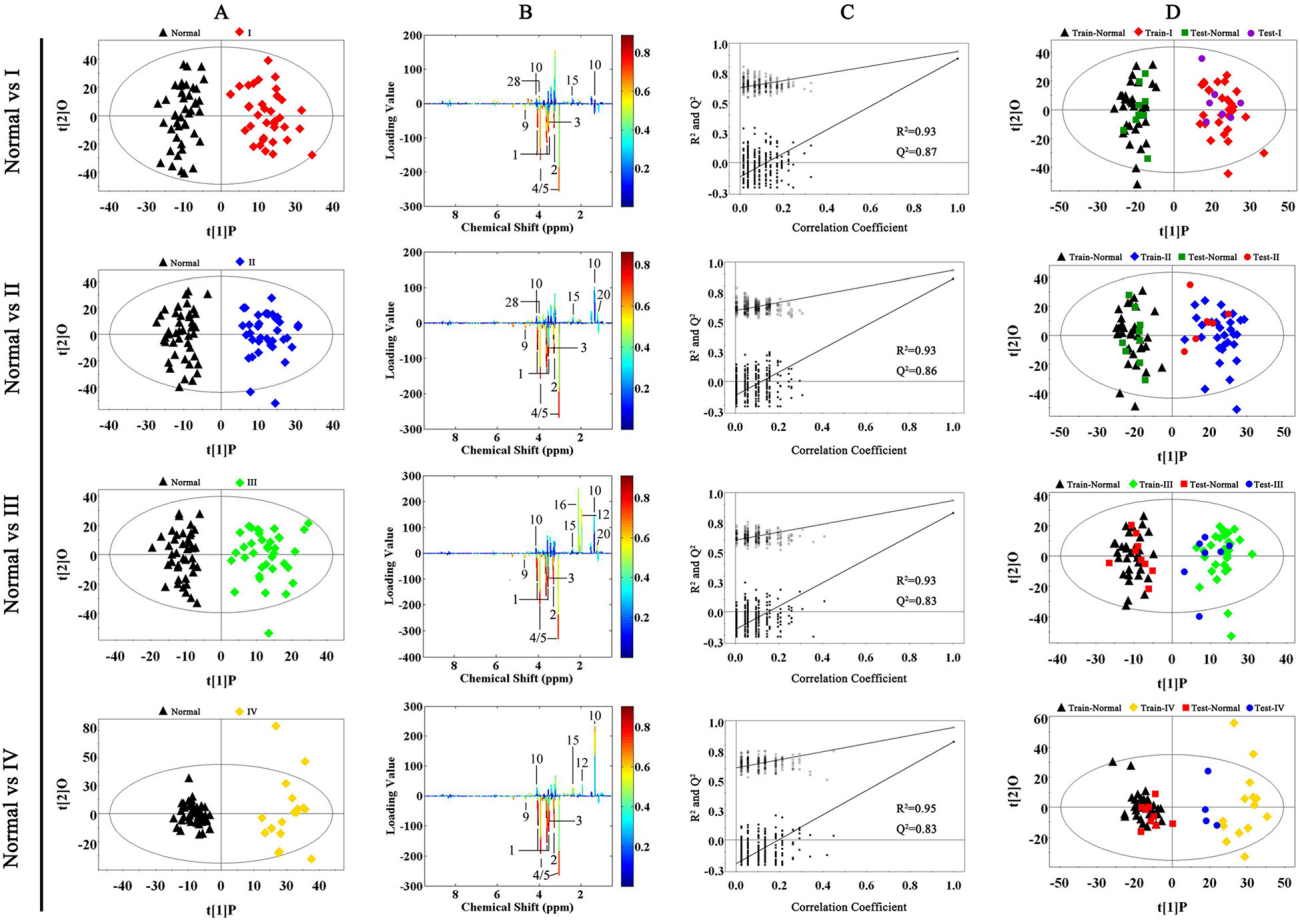
**Metabolite profiles between different stages of rectal cancer tissues and normal controls. A** OPLS-DA scores plots based on each stages of rectal cancer tissues and normal controls; black triangles represent normal controls (n = 43); red diamonds represent stage I (n = 35); blue diamond’s represent stage II (n = 37); green diamonds represent stage III (n = 37); yellow diamonds represent stage IV (n = 18). **B** Color map showed the significance of metabolite variations between the classes. Peaks in the positive direction indicated the increased metabolites in rectal cancer tissues. Decreased metabolites in rectal cancer tissues were presented as peaks in the negative direction. **C** Statistical validation of the corresponding PLS-DA models using permutation analysis (200 times). R^2^ is the explained variance, and Q^2^ is the predictive ability of the model. **D** Scores plots of OPLS-DA prediction model. 80% of samples were applied to construct the model, and then used it to predict the remaining 20% of samples.

The differences of metabolic profiling among various stages of rectal cancer are important for biomarker identification and for accurate molecular diagnosis and therapy. OPLS-DA analysis was applied to distinguish the metabolites difference between normal controls and each stage of rectal cancer tissues. The scores plots of PC1 and PC2 showed that all stages (I, II, III and IV) of rectal cancer tissues could be clearly distinguished from normal controls (Figure 3A). A panel of 40 metabolites with VIP > 1 from the training set and p < 0.05 from Student’s t-test were identified and summarized (Table 2). As shown in Table 2, creatine, uracil, succinate and β-hydroxybutyrate changed along with the process of the rectal cancer. Valine, lactate, glutamine, alanine, trimethylamine-n-oxide (TMAO), lysine and PC (phosphocholine) were increased in all rectal cancer patients except stage I. Interestingly, acetate, o-acetyl glycoprotein, dimethylamine and leucine became significantly different form stage III to stage IV. Moreover, NAD, formic acid, acetone, isoleucine, acetoacetic acid, sarcosine and acetoacetate were significantly up-regulated only in stage IV in comparison with normal controls. The corresponding loading plots based on OPLS-DA models were presented in Figure 3B. The color scale corresponded to the UV model variable weights. The relative changes in metabolites with significant correlation coefficients were a major discriminating factor among different populations, implying the biochemical alterations in different morbidity.

**Table 2:** Metabolite changes between each stage of rectal cancers and normal controls

**Metabolites**	**chemical shift**	**Mutiplicity**^**a**^	**Normal control vs. I**			**Normal control vs. II**			**Normal control vs. III**			**Normal control vs. IV**		
	**(ppm)**		**VIP**^**b**^	***P*-value**^**c**^	**FC**^**d**^	**VIP**^**b**^	***P*-value**^**c**^	**FC**^**d**^	**VIP**^**b**^	***P*-value**^**c**^	**FC**^**d**^	**VIP**^**b**^	***P*-value**^**c**^	**FC**^**d**^
Myo-inositol	4.06	t	3.04	<0.001	-1.90	3.35	<0.001	-1.93	3.99	<0.001	-2.17	3.16	<0.001	-2.07
	3.55	dd	2.92	<0.001	-1.95	3.27	<0.001	-1.99	3.89	<0.001	-2.20	3.09	<0.001	-2.08
	3.63	t	2.35	<0.001	-2.00	2.71	<0.001	-2.04	3.42	<0.001	-2.36	2.80	<0.001	-2.42
Taurine	3.27	t	3.10	<0.001	-2.01	3.37	<0.001	-2.03	3.99	<0.001	-2.28	3.18	<0.001	-2.10
α-Glucose	3.55	dd	2.92	<0.001	-1.95	3.27	<0.001	-1.99	3.89	<0.001	-2.20	3.09	<0.001	-2.08
	5.23	dd	2.60	<0.001	-4.75	2.81	<0.001	-5.58	3.07	<0.001	-4.57	2.44	<0.001	-4.97
Phosphocreatine	3.93	s	3.02	<0.001	-2.34	3.10	<0.001	-2.07	3.50	<0.001	-2.09	2.91	<0.001	-2.02
	3.04	s	2.74	<0.001	-2.29	2.94	<0.001	-2.15	3.36	<0.001	-2.16	2.66	<0.001	-1.71
Creatine	3.94	s	3.02	<0.001	-2.34	3.10	<0.001	-2.07	3.50	<0.001	-2.09	2.91	<0.001	-2.02
	3.04	s	2.74	<0.001	-2.29	2.94	<0.001	-2.15	3.36	<0.001	-2.16	2.66	<0.001	-1.71
Glycolate	3.93	s	3.02	<0.001	-2.34	3.10	<0.001	-2.07	3.50	<0.001	-2.09	2.91	<0.001	-2.02
Betaine	3.89	s	1.97	<0.001	-1.85	2.56	<0.001	-2.10	2.57	<0.001	-1.88	1.88	<0.001	-1.62
Dimethylglycine	3.71	s	2.21	<0.001	-2.58	2.33	<0.001	-2.60	2.39	<0.001	-2.13	1.98	<0.001	-2.22
Glyceryl	4.3	m	2.32	<0.001	-1.48	2.40	<0.001	-1.46	2.59	<0.001	-1.45	1.98	<0.001	-1.38
Lactate	1.33	d				1.23	<0.001	1.27	1.84	<0.001	1.26	2.26	<0.001	1.22
	4.11	q				1.13	0.004	1.73	1.52	0.001	1.88	1.52	0.001	2.22
Threonine	1.33	d	0.08	<0.001	1.21	1.23	<0.001	1.27	1.84	<0.001	1.26	2.26	<0.001	1.22
Acetate	1.93	s							2.65	<0.001	6.95	1.61	<0.001	2.96
Glutathione	2.56	m	2.49	<0.001	1.62	2.41	<0.001	1.60	2.30	<0.001	1.46	2.35	<0.001	1.81
	2.96	m							1.84	<0.001	1.42	1.50	<0.001	1.39
Uracil	7.54	d	1.99	<0.001	3.04	2.58	<0.001	3.69	2.33	<0.001	2.94	2.33	<0.001	2.46
	5.8	d	0.91	<0.001	3.06	1.84	<0.001	3.60	2.12	<0.001	3.45	2.19	<0.001	2.67
Succinate	2.41	s	1.05	0.004	1.48	1.15	0.005	1.68	1.93	<0.001	1.75	2.08	<0.001	3.03
O-acetyl glycoprotein	2.07	s							2.30	<0.001	2.65	1.30	0.033	1.35
Mannitol	3.69	m	1.31	0.015	-1.20	1.79	<0.001	-1.32	1.53	0.005	-1.23	1.48	<0.001	-1.46
	3.88	m	1.44	<0.001	-1.31	1.68	<0.001	-1.36	1.37	0.004	-1.25	1.04	0.026	-1.24
Dimethylamine	2.73	s							1.24	0.069	1.16	1.97	<0.001	2.32
Leucine	0.96	t							1.22	0.004	1.19	1.73	<0.001	1.30
Valine	1.05	d										1.47	<0.001	3.29
	0.99	d				1.84	0.011	1.31	1.59	0.023	1.37	1.46	<0.001	1.93
β-hydroxybutyrate	1.2	d	1.10	0.613	-1.07	1.55	<0.001	2.75	2.49	<0.001	2.68	1.27	0.010	1.53
Glutamine	2.14	m							1.34	0.0011	1.50	1.43	0.003	1.75
	3.77	m				1.91	<0.001	1.53	1.43	<0.001	1.31	0.87	0.097	1.18
Acetoacetate	2.28	s										1.90	<0.001	1.55
Sarcosine	2.75	s										1.58	<0.001	2.15
Tyrosine	7.2	d	1.74	0.016	-1.38	1.64	0.012	-1.43	1.42	0.051	-1.28	1.31	0.004	-1.95
	6.9	d	1.61	0.277	-1.21	1.40	0.275	-1.20	1.29	0.269	-1.21	1.22	0.006	-2.55
Alanine	1.48	d				1.69	0.010	1.59	1.59	0.014	1.81	1.00	0.022	2.79
Acetoacetic acid	2.31	s										1.77	<0.001	1.60
Serine	3.98	m	1.31	<0.001	1.49	1.93	<0.001	1.56	1.58	<0.001	1.38	1.63	0.004	1.29
Isoleucine	1.01	d										1.16	0.006	1.54
	0.95	t										1.46	0.019	1.21
Trimethylamine-N-oxide	3.27	s				1.60	<0.001	3.59	1.23	<0.001	2.73	1.67	0.008	2.09
Lysine	3.77	m				1.91	<0.001	1.53	1.43	<0.001	1.31			
Acetone	2.23	s										1.34	0.003	1.49
PC(phosphochline)	3.21	s				1.68	<0.001	1.31	1.13	0.017	1.19	1.40	0.003	1.22
GPC(glycerophosphochline)	3.23	s	2.00	<0.001	1.49	1.75	<0.001	1.51	1.14	0.002	1.26	1.92	<0.001	1.35
NAD	8.83	d										1.05	0.015	3.25
	9.15	d										1.55	<0.001	1.97
2-Hydroxyisobutyric acid	1.44	s	1.00	0.018	-1.18	1.17	0.007	-1.22	1.23	0.012	-1.20			
Trytophan	7.29	m	1.35	0.012	-1.32	1.75	<0.001	-1.50						
Formic acid	8.44	s										1.53	<0.001	1.93

^a^Multiplicity: *s* singlet, *d* doublet, *t* triplet, *q* quartet, *dd* doublet of doublets, *m* multiplet.

^b^Variable importance in the projection was obtained from OPLS-DA model with a threshold of 1.0.

^c^*p*-value obtained from Student’s t-test.

^d^Fold change(FC) between rectal cancers and normal controls. Fold change with a positive value indicates a relatively higher concentration present in rectal cancer patients while a negative value means a relatively lower concentration as compared to the normal controls.

Model parameters of permutation analysis for different stages were as follows: stage I: R^2^ = 0.93, Q^2^ = 0.87; stage II: R^2^ = 0.93, Q^2^ = 0.86; stage III: R^2^ = 0.93, Q^2^ = 0.83 and stage IV: R2 = 0.95, Q2 = 0.83. These parameters indicated the excellence of the model (Figure 3C). To further confirm the performance of these models, 80% of samples were randomly selected as training samples. Prediction parameters of the remaining 20% of samples using OPLS-DA model established with the training samples: Normal vs stage I: R^2^X_cum_ = 0.179, R^2^Y_cum_ = 0.928, Q^2^Y_cum_ = 0.828; Normal vs stage II: R^2^X_cum_ = 0.19, R^2^Y_cum_ = 0.92, Q^2^Y_cum_ = 0.829; Normal vs stage III: R^2^X_cum_ = 0.16, R^2^Y_cum_ = 0.946, Q^2^Y_cum_ = 0.81; Normal vs stage IV: R^2^X_cum_ = 0.16, R^2^Y_cum_ = 0.941, Q^2^Y_cum_ = 0.754) (Figure 3D).

### Trending biomarkers

**Figure 4:**
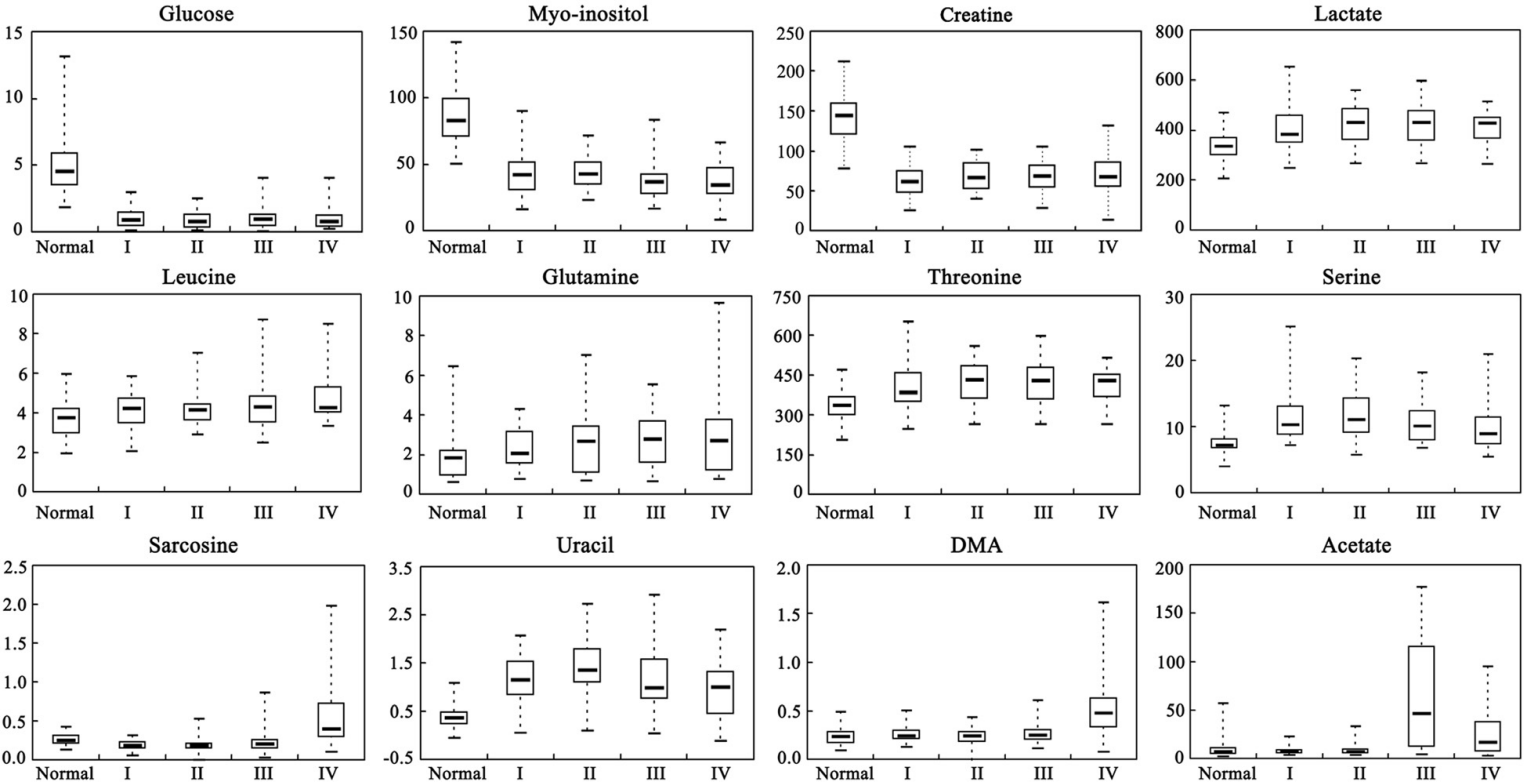
**Box-and-whisker plots illustrating discrimination between different stages of rectal cancers and normal controls.** Horizontal line in the middle portion of the box, median; bottom and top boundaries of boxes, 25th and 75th percentiles, respectively; lower and upper whiskers, 5th and 95th percentiles, respectively.

Biomarker identification is important for detecting the rectal cancer formation, invasion, and metastasis. The representative metabolites with significant difference between controls and rectal cancer tissues were represented in box-and-whisker plots (Figure 4), which showed the concentration ranges, median quartiles and extremes.

The decrease of glucose and increase of lactate in tumor tissues was not surprising because of the Warburg effect. The results of changes in myo-inositol and glucose in our work were consistent with a previous research on breast cancer [16]. Myo-inositol, a precursor in the phosphatidylinositol cycle and a source of several second messengers, was decreased along with the progression of rectal cancers compared with normal controls. The function of myo-inositol as an osmoregulator in different stages of malignant transformation could be a further explanation for our results.

Many studies have found free amino acids altered in patients with different kinds of cancer [14,17]. In our study, leucine, glutamine, threonine and serine were significantly increased along with the progression of rectal cancer, which can be explained as cellular needs for higher turnover of structural proteins in cell proliferation. Sarcosine, an N-methyl derivative of the amino acid glycine, was significantly up-regulated in stage IV. Uracil is an indicator of transcription, whose increase suggests cell proliferation up-speeded. Methylamine, DMA and TMAO, the products of choline metabolism, were also altered in our study,indicating the disturbance of choline metabolism.

**Figure 5:**
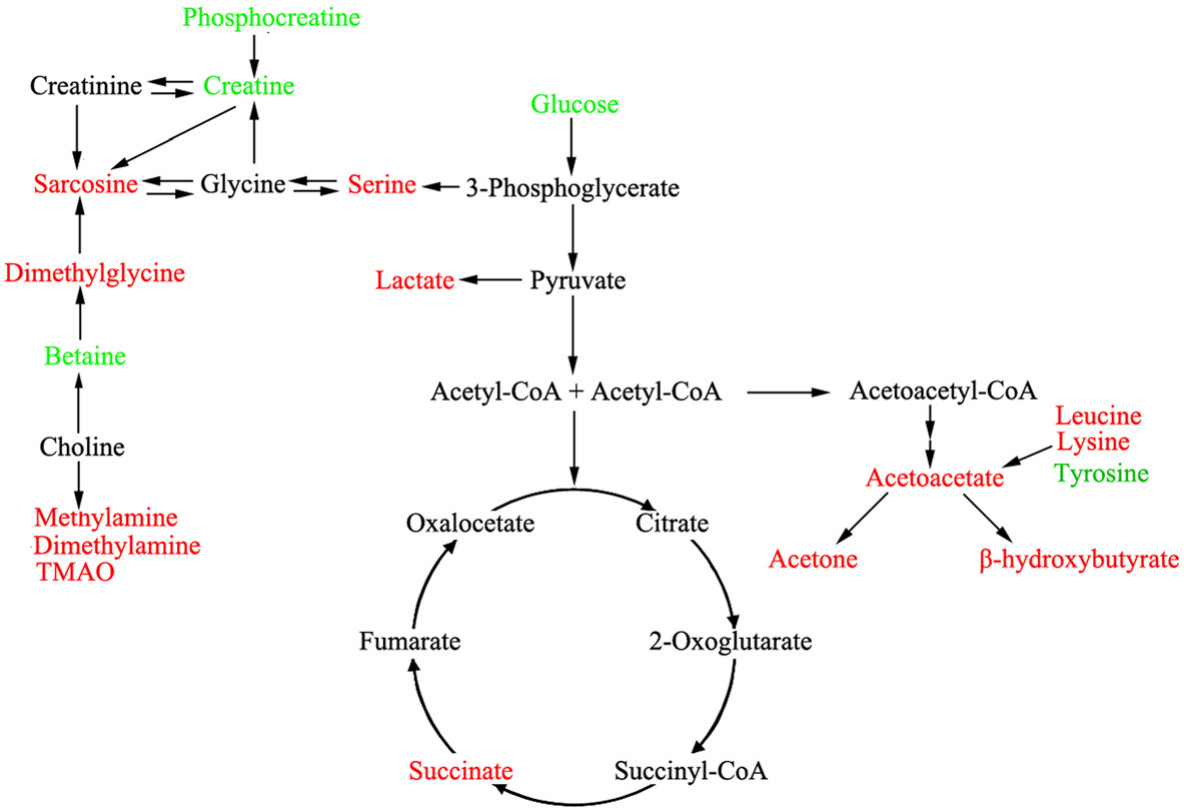
**Disturbed metabolic pathways of the most relevant metabolites between rectal cancers and normal controls.** Green: lower concentration in rectal cancer patients than in normal controls. Red: higher concentration in rectal cancer patients than in normal controls.

Based on the modified metabolites, we summarized a related metabolic pathway of rectal cancer. As shown in Figure 5, the disturbed metabolic pathway included glycolysis (glucose, lactate), tricarboxylic acid cycle (succinate), choline metabolism (TMAO, DMA, methylamine, betaine, dimethylgcine, sarcosine, creatine and phosphocreatine), ketone body (acetoacetate, β-hydroxybutyrate and acetone) and amino acid metabolism (serine and glycine).

## Discussion

In present study, we investigated the metabolic profiling of human rectal cancer tissue based on ^1^H NMR. Forty distinguishing metabolites were identified, 16 of which significantly changed along with the progression of rectal cancer. These modified metabolites consisted of lactate, threonine, acetate, glutathione, uracil, succinate, serine, formate, lysine, tyrosine, myo-inositol, taurine, phosphocreatine, creatine, betaine and dimethylglycine. Though there was one report showing the metabolic profiling of colorectal cancer tissues [1], to the best of our knowledge, the present study is the first to show the distinguishing metabolites in human rectal cancer tissues. More importantly, we identified the specific metabolites changed along with process of rectal cancer. Compared with Keun’s study in metabolic profiling of human colorectal cancer tissues, 16 metabolites correlated with the stage of rectal cancer were newly identified except lactate and taurine. The large cohort of tissue samples and samples coming from same kind of tissue might be the most important reason that we identified more new metabolites.

Metabolites identification is critically important to understand the potential biological alterations associated with rectal cancer morbidity and to facilitate this metabolic approach into clinical use. Based on ^1^H NMR, glucose was apparently lowered in rectal cancer tissues whereas lactate and serine were consistently elevated. The results were not unexpected because of Warburg effect. Cancer cells prefer to metabolize glucose through glycolysis to generate ATP instead of oxidative phosphorylation even in presence of ample oxygen [18]. This process is less efficient because one molecule of glucose just generates 2 molecules ATP by glycolysis instead of 36 molecules through oxidative phosphorylation. Thus, cancer cells enhance glucose uptake to meet the energy requirement of maintaining their quick proliferation. Along with the decrease of glucose, the end product of glycolysis, lactate is found to accumulate in rectal cancer tissues. Lactate is able to make the extracellular pH of the tumor consistently acidic which would stimulate tumor cell invasion in vitro and metastasis in vivo [15,19]. Our results were consistent with previous reports that the decrease of glucose and increase of lactate were also observed in stomach cancer, oral cancer etc. [20,21].

In addition to increased glycolysis, a higher level of serine in rectal cancer tissues was also observed. Recently, Oliver et al. reported that human cancer cells rapidly used exogenous serine and serine deprivation triggered activation of serine synthesis pathway, resulting in an increased flux to tricarboxylic acid cycle [22]. Serine is generated from the glycolytic intermediate 3-phosphoglycerate generated by 3-phosphoglycerate dehydrogenase (PHGDH). PHGDH is recurrently amplified in a genomic region of focal copy number gain in melanoma based on an analysis of human cancers [23]. Reducing PHGDH expression impairs the proliferation in amplified cell, whereas overexpression of PHGDH in human breast cancer contributed to carcinogenesis by facilitating glycolytic pathway to serine biosynthetic pathway [24]. These observations together with our findings strongly support a notion that altered serine metabolism leads disturbance in human rectal cancer.

In our study, phosphocreatine, creatine, dimethylgcine and betaine were down-regulated in rectal cancer tissues, and the levels of methylamines (methylamine, DMA, TMAO) and sarcosine were obviously increased. These metabolites are all involved in choline metabolism pathway. Choline and its derivatives are important constituents in phospholipid metabolism of cell membranes and identified as markers of cell proliferation. Although methylamines, products of choline metabolism, are usually regarded as nontoxic substances, they could induce hepatocarcinogenesis in rats and the similar mechanism may exist in human [25]. Therefore, methylamines may indicate the disturbance of liver homeostasis in development of rectal cancer. The creatine/creatine kinase (CK)/phosphocreatine system plays a key role in cellular energy buffering and transport, especially in cells with high and disturbed energy metabolism. Moreover, creatine shows significant anticancer effect against brain tumor, oral squamous cell carcinoma and childhood cerebellar tumour [20,26,27]. Sarcosine, a metabolite in choline metabolism pathway, is generated from glycine by glycine-N-methyl transferase. In the present study, sarcosine was significantly enhanced, especially in stage IV of rectal cancer tissues.

Ketone bodies (KB), including acetoacetate (AcAc), β-hydroxybutyrate (βOHB) and acetone, are important metabolic substrates. They are produced by liver under conditions of fasting and caloric restriction. KB elevation suggests that it can provide more energy for cell proliferation and compensate the shortage of energy. Our results showed that βOHB in rectal cancer tissues increased 2 folds in comparison to normal controls, which was consistent with a previous study that βOHB was significantly increased in breast cancer and colorectal cancer [28,29]. Moreover, we firstly found other two KB, AcAc and acetone, were also up-regulated in rectal cancer tissues, with increases of 1.2 and 1.1 folds, respectively. βOHB is a predominant KB, which belongs to energy-rich compounds transporting energy from the liver to other tissues. A previous study showed that the concentration of βOHB in blood was 3-flod higher than AcAc during fasting [30]. Based on our observation that βOHB changed more significantly than other two KB, we speculated that βOHB may play a central role in rectal cancer progression. Recently, βOHB is considered to be associated with epigenetic regulation except for energy carrier [31]. It acts as an endogenous and specific inhibitor of class I histone deacetylases, participating in regulating histone acetylation, gene expression, and eventually promoting stress resistance. Thus, βOHB up-regulation may enhance resistance of tumor tissue to damage and affect the survival of rectal cancer patients.

Some amino acid levels are reported higher in cancer tissues than in normal controls [14,21] Our results were consistent with these studies that threonine, leucine, valine, glutamine, alanine, serine, isoleucine and lysine were markedly increased in rectal cancer tissues. The accumulation of amino acids in cancer cell could be attributed to the uptake by cancer cells from normal organ and blood through the up regulation of amino acid transporters [32]. The disturbances of above amino acids reflect cellular needs for higher turnover of structural proteins in cancer cell proliferation. Uracil, an alternate of thymine, is incorporated into ribonucleic acid in transcription. We found that the level of uracil was apparently higher in the initial stages of cancer (stage I and II) and a little lower in later stages (stage III and IV), suggesting that cell proliferation may be accelerated in the process of tumor formation.

Myo-inositol is a precursor in the phosphatidylinositol cycle and a source of several second messengers. Previous studies showed that myo-inositol acts as a cancer chemoprevention agent [33,34]. In our study the decreased myo-inositol implies neoplasia in human rectal tissue.

## Conclusions

In this study, we analyzed the metabolic profiling of rectal cancer tissues in comparison with normal controls based on ^1^H NMR spectroscopy combined with multivariate statistical analysis. The metabolites distinguishing rectal cancer tissues from normal controls may be involved in monitoring the neoplasia, invasion and metastasis of tumor, and be potential biomarkers in treatment of cancer. This opened a window of opportunity to improve diagnosis and treatment of malignant tumor for surgeons and patients.

## Methods

### Study populations and sample collection

A total of 127 rectal cancer patients were recruited from West China Hospital of Sichuan University during 2009 to 2010. The patients enrolled in this study did not receive any neoadjuvant chemotherapy or radiation therapy before surgical treatment. The clinical information of patients was summarized in Table 3. The rectal cancer tissue histology, tumor grade, TNM, Duke Stages was presented in Table 3. And the nutritional status of the patients, body weight and weight loss were provided (Additional file 1: Table S1). The survival rate of patients enrolled in this study was also provided (Additional file 2: Figure S1). As shown in Table 3, the stage of all tissue specimens was determined according with the American Joint Committee on Cancer (AJCC) for rectal tumors: stage I, 35 patients; stage II, 37 patients; stage III, 37 patients; stage IV, 18 patients. The protocols outlined in the following text were approved by the Ethics Committee of West China Hospital of Sichuan University. The informed consents were obtained from all patients prior to sample collection.

**Table 3:** Clinical information of rectal cancer patients used in this study

	**Rectal cancer patients**	**Normal controls**
**Number**	127	43
**Age (median,range)**	55 28-86	56 35-85
**Male/female ration**	69/58	16/27
**Histology**	Adenocarcinoma(127)	∕
**Pathologic grade**		∕
PD	36	
MD	80	
WD	7	
NA	4	
**Cancer stage/Duke**		∕
I/A(35)	T1N0M0(10)	
	T2N0M0(25)	
II/B(37)	T3N0M0(37)	
III/C(37)	T2N1aM0(3)	
	T2N2aM0(2)	
	T3N1aM0(16)	
	T3N1bM0(8)	
	T3N2aM0(4)	
	T3N2bM0(4)	
IV/D(18)	T3N0M1a(1)	
	T3N1aM1a(1)	
	T3N1bM1a(5)	
	T3N2aM1a(5)	
	T3N2bM1a(5)	
	T4aN2bM1a(1)	
**Metastatic site**		∕
I	35(0)	
II	37(0)	
III	37(lymph node)	
IV	18(liver)	

*PD* poorly differentiated.

*MD* moderately differentiated.

*WD* well-differentiated.

*NA* not applicable.

Tumor specimens and adjacent normal-appearing tissues at least 5–10 cm away from the edges of a tumor were collected from rectal cancer patients undergoing colorectal resection according to procedure reported previously [35]. In total, 170 tissue samples were obtained from patients. The tissues dissected by a senior pathologist in the operating room were immediately frozen in liquid nitrogen and stored at -80°C. The clinical diagnosis, tumor stage, histology differentiation and resection margin were determined by routine histopathology examination of H & E stained specimens by a blinded pathologist.

### Sample preparation

The frozen tissue samples ranged from 150 to 400 mg were weighed and suspended in methanol (4 ml per gram of tissue) and double distilled water (0.85 ml per gram of tissue). After vortex, chloroform (2 ml per gram of tissue) was added, followed by addition of 50% chloroform (2 ml per gram of tissue). The suspension was left on ice for 30 min, and centrifugated at 1,000 g for 30 min at 4°C. This procedure separated suspension into three phases: a water phase at the top, a denatured proteins phase in the middle, and a lipid phase at the bottom. The upper phase (aqueous phase) of each sample was collected and evaporated to dryness under a stream of nitrogen. The residue was reconstituted with 580 μl of D_2_O containing 30 μM phosphate buffer solution (PBS, pH = 7.4) and 0.01 mg/ml sodium (3-trimethylsilyl)-2, 2, 3, 3-tetradeuteriopropionate (TSP) as a chemical shift reference (δ0.0). After centrifuged at 12,000 g for 5 min, the 550 μl supernatant was transferred into a 5-mm NMR tube for NMR spectroscopy [36].

### ^1^H-NMR spectroscopic analysis

All tissue samples were analyzed by ^1^H-NMR spectroscopy at 300 K using a Bruker Avance II 600 spectrometer operating (Bruker Biospin, Germany) at 600.13 MHz. A one-dimensional spectrum was acquired by using a standard (1D) Carr-Purcell-Meiboom-Gill (CPMG) pulse sequence to suppress the water signal with a relaxation delay of 5 sec. Sixty-four free induction decays (FIDs) were collected into 64 K data points with a spectral width of 12,335.5-Hz spectral, an acquisition time of 2.66 sec, and a total pulse recycle delay of 7.66 sec. The FIDs were weighted by a Gaussian function with line-broadening factor -0.3 Hz, Gaussian maximum position 0.1, prior to Fourier transformation [37].

### Pattern recognition (PR) analysis

The raw NMR data has been manually Fourier transformed using MestReNova-6.1.1-6384 software before data processing. All of the ^1^H NMR spectra were corrected for phase and baseline distortions using MestReNova-6.1.1-6384 software. ^1^H NMR spectra of tissue samples were referenced to the TSP resonance at δ0.0. The spectrum ranging from 9.5 to 0.5 ppm was divided into 4500 integral segments of equal length (0.002 ppm). The area under the spectrum was calculated for each segmented region and expressed as an integral value. The region 4.9–4.6 ppm was removed for excluding the effect of imperfect water signal. Moreover, the integrated data were normalized before multivariate statistical analysis to eliminate the dilution or bulk mass differences among samples due to the different weight of tissue, and to give the same total integration value for each spectrum.

For multivariate statistical analysis, the normalized NMR data were imported into SIMCA-P + 11 (Umetrics, AB). The principal component analysis (PCA) was initially applied to analyze the NMR spectral data to separate the tumor samples from the normal samples. The data were visualized using the principal component (PC) score plots to identify general trends and outliers. Orthogonal projection to latent structure with discriminant analysis (OPLS-DA) was subsequently used to improve the separation. R^2^ and Q^2^ values were used to assess the amount of variation represented by the principal components and robustness of the model, respectively. The PLS-DA models were cross-validated by a permutation analysis (200 times) [38,39]. The default 7-round cross-validation was applied with 1/seventh of the samples being excluded from the mathematical model in each round, in order to guard against over fitting. The model coefficients locate the NMR variables associated with specific interventions as *y* variables. The model coefficients were then back-calculated from the coefficients incorporating the weight of the variables in order to enhance interpretability of the model: in the coefficient plot, the intensity corresponds to the mean-centered model (variance) and the color-scale derives from the unit variance-scaled model (correlation). The coefficient plots were generated with Matlab scripts with some in-house modifications and were color-coded with the absolute value of coefficients (*r*) [40,41].

To identify the variables contributed to the assignment of spectra between tumor tissues and normal controls, the variable importance in the projection (VIP) values of all peaks from OPLS-DA models was analyzed, and variables with VIP > 1 were considered relevant for group discrimination. Moreover, unpaired Student’s t-test (*p* < 0.05) to the chemical shifts was also used to assess the significance of each metabolite. Only both VIP > 1 of multivariate and *p* < 0.05 of univariate statistical significance were identified as distinguishing metabolites. The corresponding chemical shift and multiplicity of the metabolites were identified by comparisons with the previous literatures and the Human Metabolome Database (http://www.hmdb.ca/), a web-based bioinformatics/cheminformatics resource with detailed information about metabolites and metabolic enzymes.

## Competing interests

The authors declare that they have no competing interests.

## Authors’ contributions

Conception and design: HW, YLZ. Financial support: YLZ. Collection and process of samples: HW, LW, JC, BZ, PX, TW, XS, YL, ZZ. Collection and analyze the ^1^H NMR data: HW, PD. Manuscript writing: HW, YLZ. Perform the multivariate analysis and drafte: HW, HZ, JZ. All of authors read and approved the final manuscript.

## Supplementary Material

Additional file 1: Table S1 The added clinical information for Rectal cancer patients used in this study. Weight and height were then used to calculate body mass index (BMI: weight [kg]/height [m2]), which was further categorized according to the World Health Organization’s age- and sex-adjusted criteria. BMI < 18.5: undernourished; 18.5 < BMI < 24.9: normal weight; 25 < BMI < 29.9: overweight; BMI > 30: obese. Weight loss was defined as loss of more than 5% pre-illness weigh. Click here for file

Additional file 2: Figure S1 The survival rate of patients enrolled in this study until Aug, 2013. Initial stages: stage I and II; later stages: stage III and IV. Click here for file
