# Supplementary material for: ¹H NMR-based metabolic profiling of human rectal cancer tissue
Source: Mol Cancer. 2013 Oct 18;12:121. doi: 10.1186/1476-4598-12-121 (PMC3819675; doi:10.1186/1476-4598-12-121)
Supplement: Additional file 1: Table S1 — The added clinical information for Rectal cancer patients used in this study. Weight and height were then used to calculate body mass index (BMI: weight [kg]/height [m2]), which was further categorized according to the World Health Organization’s age- and sex-adjusted criteria. BMI < 18.5: undernourished; 18.5 < BMI < 24.9: normal weight; 25 < BMI < 29.9: overweight; BMI > 30: obese. Weight loss was defined as loss of more than 5% pre-illness weigh. [file 1476-4598-12-121-S1.docx]

**Additional 1: Table S1. The added clinical information of rectal cancer patients used in this study**

|  |  | Rectal cancer patients |
| --- | --- | --- |
| **Number** |  | 127 |
| **Weight (kg, median,range)** |  | 57 35-87 |
| **Weight loss** |  |  |
| 0 |  | 86 (67.7%) |
| ﹤5% |  | 21 (16.5%) |
| ≥5% |  | 20 (15.8%) |
| **BMI (kg/m^2^)** |  |  |
| BMI<18.5 |  | 8 (6.3%) |
| 18.5<BMI<24.9 |  | 103 (81.1%) |
| 25<BMI<29.9 |  | 15 (11.8%) |
| BMI>30 |  | 1 (0.8%) |
