# Supplementary figures and images for: ¹H NMR-based metabolic profiling of human rectal cancer tissue
Source: Mol Cancer. 2013 Oct 18;12:121. doi: 10.1186/1476-4598-12-121 (PMC3819675; doi:10.1186/1476-4598-12-121)

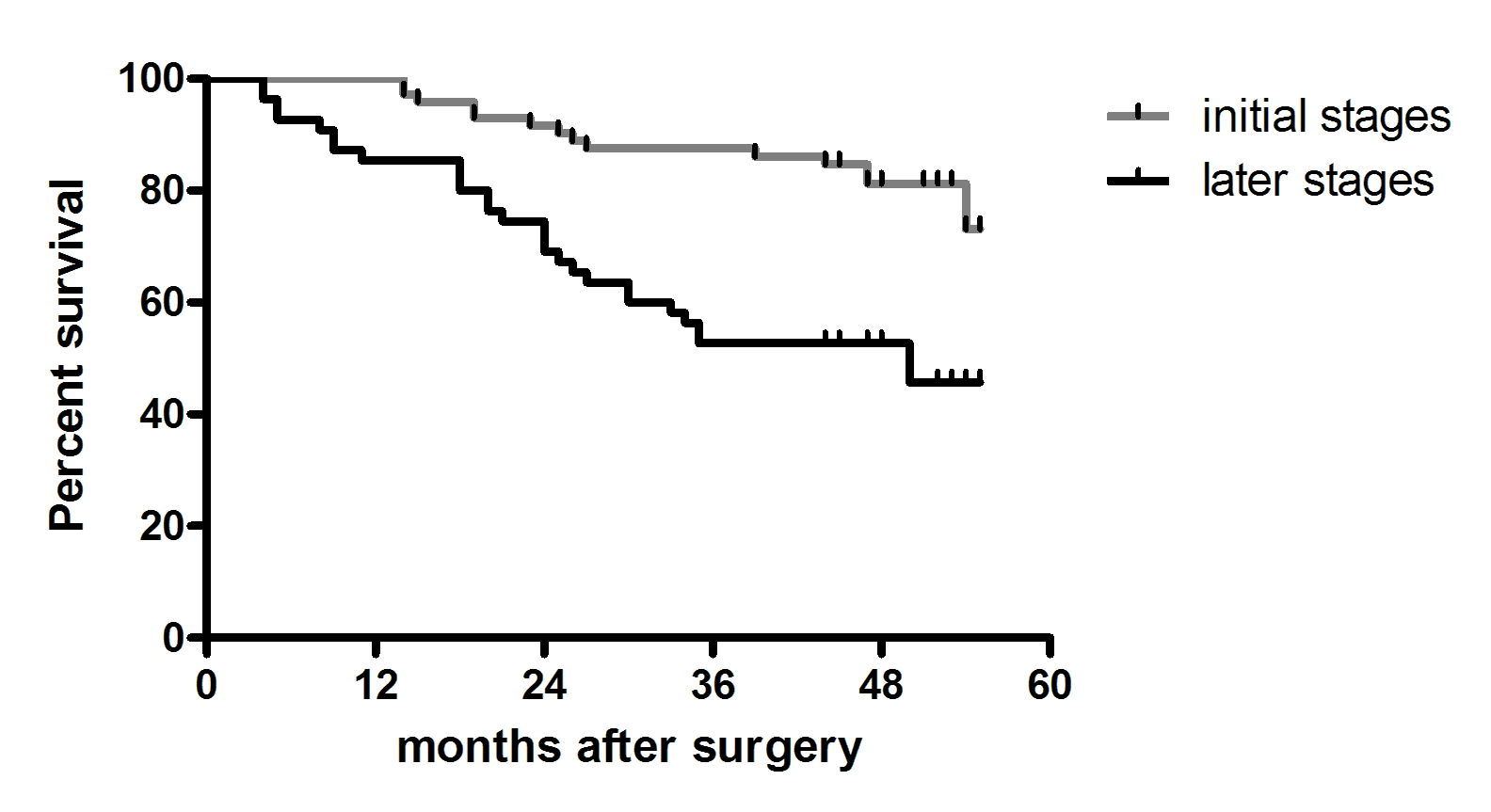

Supplement: Additional file 2: Figure S1 — The survival rate of patients enrolled in this study until Aug, 2013. Initial stages: stage I and II; later stages: stage III and IV. [file 1476-4598-12-121-S2.tiff]
